# Correlation between extent of lacrimal gland prolapse and clinical features of thyroid-associated ophthalmopathy: a retrospective observational study

**DOI:** 10.1186/s12886-022-02270-9

**Published:** 2022-02-10

**Authors:** Yang Gao, Qinglin Chang, Yang Li, Hanqiao Zhang, Zhijia Hou, Zheng Zhang, Zheng Li, Dongmei Li

**Affiliations:** 1 Department of Ophthalmology, Beijing Tongren Eye Center, Beijing Ophthalmology & Visual Sciences Key Lab, Beijing Tongren Hospital, Capital Medical University, Beijing, China; 2 Department of Radiology, Beijing Tongren Hospital, Capital Medical University, Beijing, China

**Keywords:** Proptosis, Extraocular muscles, Lacrimal gland, Magnetic resonance imaging, Thyroid-associated ophthalmopathy, Graves’ disease, Prolapse

## Abstract

**Background:**

Studies on the factors related to lacrimal gland prolapse (LGP) in patients with thyroid-associated ophthalmopathy (TAO) are limited. This study aimed to assess the factors associated with LGP on magnetic resonance imaging (MRI) and its relation to TAO activity .

**Methods:**

Thirty-six patients (72 orbits) with inactive TAO (43 orbits, Clinical Activity Score [CAS] < 3) or active TAO (29 orbits, CAS ≥3) were investigated retrospectively. All patients underwent ophthalmic evaluation and orbital magnetic resonance imaging. The severity of LGP and proptosis and the extraocular muscle (EOM) volume were measured. LGP and related factors were assessed by correlational and linear regression analyses. The value of LGP for discriminating the activity of TAO was evaluated by receiver-operating characteristic curve analysis.

**Results:**

The mean LGP was significantly higher in the active TAO group than in the inactive TAO group (*P* < 0.001). There were significant positive correlations between LGP severity and the CAS (*r* = 0.51, *P* < 0.001), proptosis (*r* = 0.72, P < 0.001), and EOM volume (superior rectus [r = 0.49, *P* < 0.001], inferior rectus [*r* = 0.47, *P* < 0.001], lateral rectus [*r* = 0.59, *P* < 0.001], medial rectus [*r* = 0.62, *P* < 0.001], superior oblique [*r* = 0.48, *P* < 0.001], and all EOMs [*r* = 0.59, *P* < 0.001]). Receiver-operating characteristic curve analysis revealed an LGP of 13.65 mm (area under the curve, 0.824; sensitivity, 79.3%; specificity, 81.4%) to be the cut-off value that differentiated active and inactive TAO.

**Conclusions:**

LGP measurements obtained from orbital magnetic resonance images were positively correlated with CAS, proptosis and EOM volume. The extent of LGP appears to be a good indicator of disease activity in patients with TAO.

## Background

Thyroid-associated ophthalmopathy (TAO) is the most common extrathyroidal manifestation of Graves’ disease [1, 2] and is found in 25–50% of patients with this disorder [3]. Previous studies have shown that 65–85% of patients with TAO have lacrimal gland involvement, which leads to reduced tear secretion [4]. Patients typically complain of excess tearing, photophobia, grittiness, foreign body sensation, and other symptoms of xerophthalmia. Several magnetic resonance imaging (MRI) and computed tomography (CT) studies have demonstrated that the volume of the lacrimal gland is significantly larger in patients with TAO than in healthy controls [5–7]. Clinically, the lacrimal gland is not only enlarged in TAO but also prolapsed out of the orbit; however, at present, there is limited information on the factors related to lacrimal gland prolapse (LGP) in patients with TAO. Therefore, the purpose of this study was to identify a simple and convenient quantitative MRI parameter that can measure the degree of LGP and to be a good indicator of TAO activity.

## Methods

### Study subjects

Thirty-six consecutive patients with TAO who underwent MRI for assessment of disease severity in the Department of Ophthalmology at Beijing Tongren Hospital between March 2019 and May 2020 were enrolled in the study. All patients had bilateral orbital involvement.

The study inclusion criteria were as follows: fulfillment of the European Group on Graves’ Orbitopathy diagnostic criteria for TAO [8]; no prior history of immunosuppressive therapy, retrobulbar radiotherapy, or surgical decompression of the orbit; and complete orbital MRI data available for assessment. The following exclusion criteria were applied: other disease involving the intraorbital structures regardless of the etiology, other autoimmune disease, any ferromagnetic or electronic material in the body, and claustrophobia.

A modified version of the Clinical Activity Score (CAS) [9] was used to assess the activity of TAO. The CAS includes scores for the following seven items: pain on attempted up or down gaze, spontaneous retrobulbar pain, redness of the conjunctiva, inflammation of the caruncle and/or plica, redness of the eyelids, swelling of the eyelids, and conjunctival edema. We obtained the CAS for each eye. Based on the CAS, the 72 eyes of the 36 patients were categorized into an inactive TAO group (43 orbits, CAS < 3) and an active TAO group (29 orbits, CAS ≥3).

Previous researchers found no significant difference in lacrimal gland parameters measured for the left and right sides in patients with TAO [10]. Therefore, in this study, we combined the measurements for the left and right eyes for the analyses.

### MRI examinations

Orbital MRI scans were obtained for all patients using a 3.0-T scanner (Signa HDx; General Electric Healthcare, Milwaukee, WI, USA) with an eight-channel phased-array head coil. Foam cushions were placed between the head and the coil to stabilize the head in the supine position. Throughout the scanning procedure, patients needed to remain stationary and keep their eyes closed to minimize the impact of ocular movements.

The orbital MRI protocol involved both sagittal and axial turbo spin echo (TSE) T1-weighted imaging (T1WI), axial and coronal TSE T2-weighted imaging (T2WI), and T2WI with fat suppression (T2WI-FS). The parameters used for T1WI were as follows: an echo time (TE) of 9 ms and a repetition time (TR) of 456.5 ms. The following parameters were used for T2WI and T2WI-FS: TR/TE 1621.5 ms/90 ms, two excitations, 384 × 256 matrix, field of view 180 × 180 mm, section thickness 3 mm; and section gap 0 mm.

### Imaging processing

#### Measurement of LGP severity

**Fig. 1:**
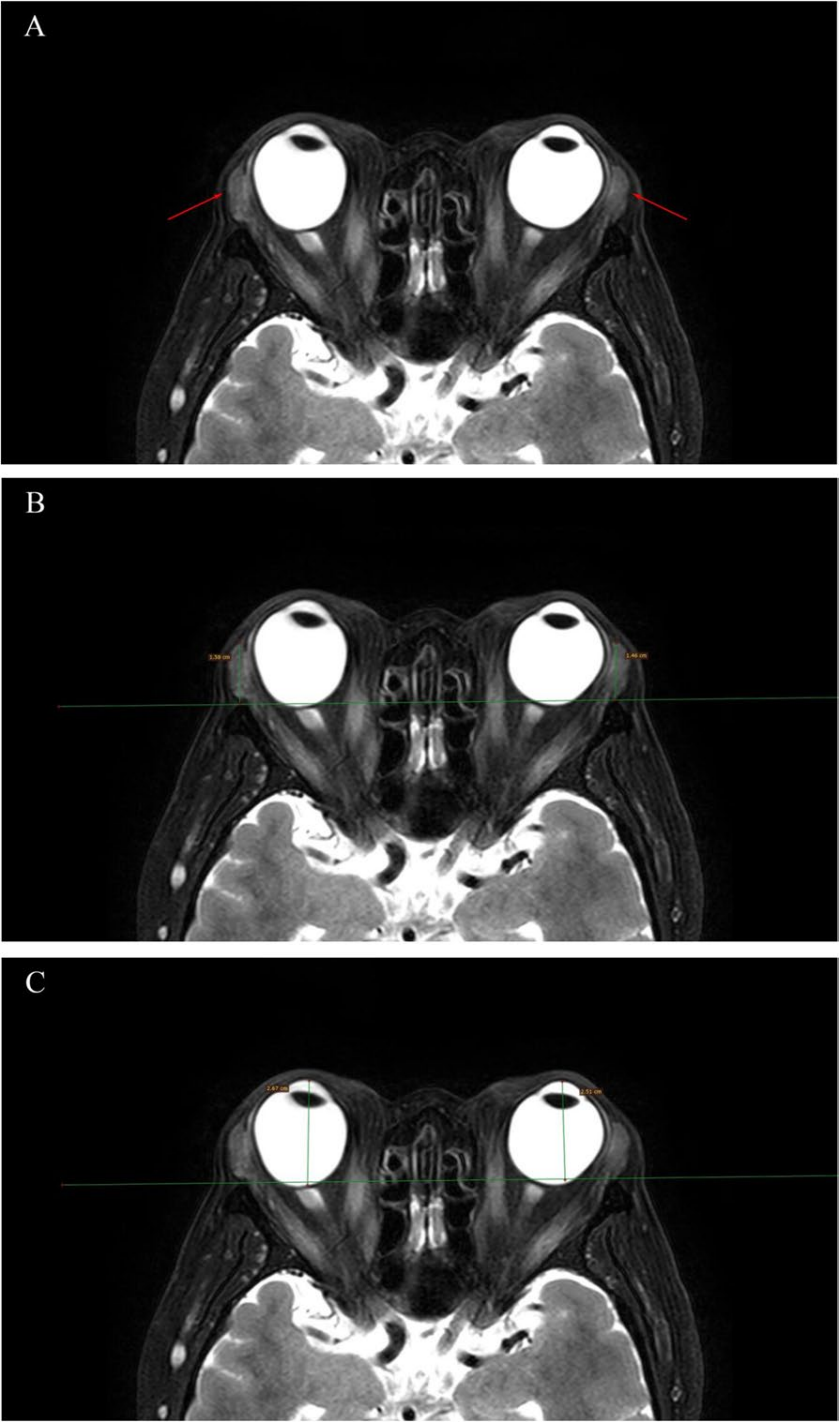
An axial 3-mm-thick T2-weighted fat suppressed axial image of a patient with thyroid-associated ophthalmopathy. The arrows in (**A**) show bilateral prolapse of the lacrimal glands. (**B**) Lacrimal gland prolapse, measured as the vertical distance from the top of the lacrimal gland to the interzygomatic line, is 15.1 mm on the right and 14.6 mm on the left. (**C**) Exophthalmos is measured as the vertical distance from the top of the cornea to the interzygomatic line

The degree of LGP was measured on axial T2WI-FS sequences using ImageJ software (National Institutes of Health, Bethesda, MD, USA, http://rsbweb.nih.gov/ij/index.html). Using the method described by Gagliardo et al. [11]^,^ a line was drawn on the maximum delineated LGP level of the ventral zygomatic border (interzygomatic line) bilaterally, after which a perpendicular line was taken to measure the degree of herniation of the lacrimal gland parenchyma (Fig. 1).

#### Measurement of proptosis

We measured the severity of proptosis on axial T2WI using ImageJ software. At the level showing the maximum convexity of the lens, the vertical distance from the interzygomatic line to the top of the cornea was defined as the proptosis value (Fig. 1).

#### Measurement of extraocular muscle volume (EOM)

**Fig. 2:**
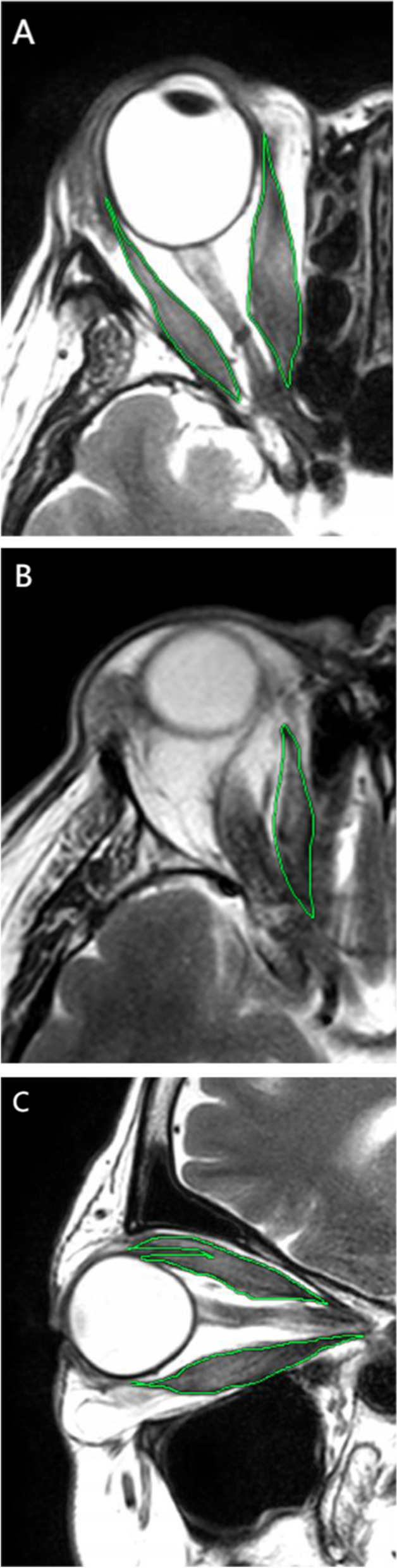
The cross-sectional area of the extraocular muscle was measured on T2-weighted magnetic resonance imaging scans. (**A**) The areas of the medial rectus and lateral rectus were obtained from axial images. (**B**) The areas of the superior oblique muscles were obtained from the axial images. (**C**) The areas of the superior rectus complex and inferior rectus were obtained from the sagittal images

The volume of each EOM measured on MRI was similar to that in previous studies [12–14]. For each orbit, the EOM volumes of the lateral rectus, medial rectus, inferior rectus, and superior oblique muscles were assessed; those for the superior rectus complex, the superior rectus and levator palpebrae muscles were measured together because they were difficult to separate on the magnetic resonance images (Fig. 2). Regions of interest were delineated using the Image J polygon selection tool and their cross-sectional areas were measured. The areas of the medial rectus, lateral rectus, and superior oblique muscles were obtained from axial images and those of the superior rectus complex and inferior rectus from the sagittal images. The EOM volumes were obtained from the sum of cross-sectional areas with a slice thickness of 3 mm. All data were determined independently in masked mode by one radiologist and one senior ophthalmologist; the average measurements were used for the statistical analyses.

### Statistical analysis

The Shapiro-Wilk test was used to evaluate the normality of continuous variables. Data are summarized as the mean ± standard deviation or as the median and interquartile range depending on whether they were continuous and normally distributed. The chi-squared test and Student’s *t*-test were used to evaluate differences in sex and age between the two groups. All quantitative measurements were compared between the two groups using the Student’s *t*-test. Linear regression analysis with Pearson’s or Spearman’s test was performed to explore the correlation between the LGP and CAS, proptosis, EOM volumes, and age in all patients with TAO.

Receiver-operating characteristic (ROC) curve analysis was used to estimate the value of LGP in discriminating active from inactive TAO. The intraclass correlation coefficient (ICC) was used to evaluate the consistency of the quantitative parameters measured by the two observers. All statistical analyses were performed using SPSS software version 19.0 (IBM Corp., Armonk, NY, USA). A *P*-value of < 0.05 was considered statistically significant.

## Results

### Baseline demographic and clinical characteristics

The study group consisted of 16 men and 20 women with an average age of 51.59 ± 12.42 years (range, 21–74). The interobserver agreement was excellent (ICC > 0.9) for all quantitative measurements. The baseline clinical characteristics of the two groups are described in detail in Table 1. There was no significant difference in sex (*P* = 0.957) or age (*P* = 0.48) between the two groups.

**Table 1:** Comparison of demographic and quantitative measurements between patients with active and inactive thyroid-associated ophthalmopathy

	Inactive group	Active group	P-value
Orbits, n	43	29	/
Age, n	51.3 ± 14.0	54.1 ± 8.4	0.48
Female/Male	24/19	16/13	0.957
CAS	1.28 ± 0.69	4.21 ± 0.80	/
Proptosis (mm)	18.83 ± 3.02	22.61 ± 4.27	<0.001
LGP (mm)	12.21 ± 2.09	15.50 ± 3.2	<0.001

*CAS* Clinical Activity Score; *LGP* lacrimal gland prolapse

### Comparison of LGP between the active and inactive TAO groups

**Fig. 3:**
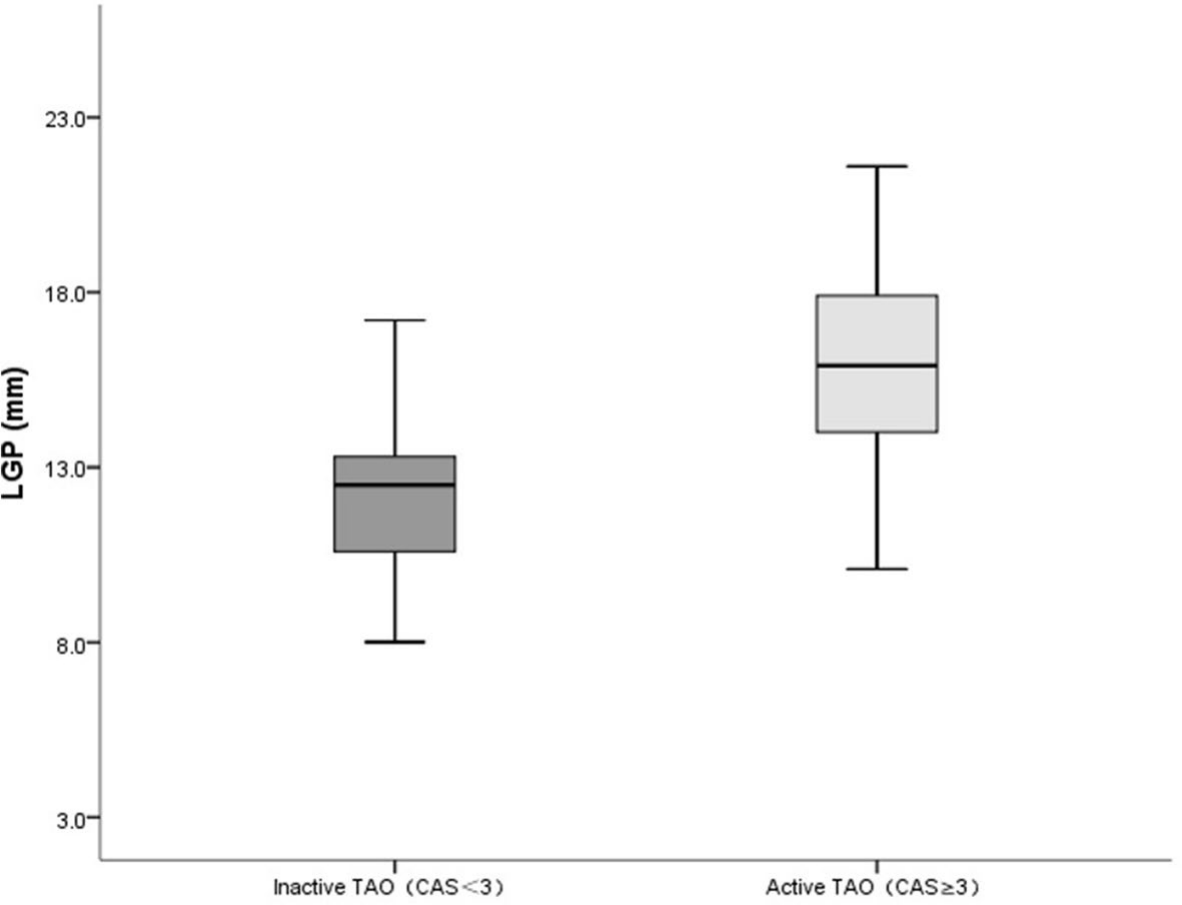
Graph comparing the degree of lacrimal gland prolapse between the group with active TAO and the group with inactive TAO. The plots and bars indicate the mean and standard deviation. The mean LGP in the active group was significantly greater than that in the inactive group (*P* < 0.001). CAS, Clinical Activity Score; LGP, lacrimal gland prolapse; TAO, thyroid-associated ophthalmopathy

The mean LGP was significantly greater in the active group than in the inactive group (15.50 ± 3.25 vs 12.21 ± 2.09 mm; *P* < 0.001) (Fig. 3).

### Correlation between LGP severity and CAS

**Fig. 4:**
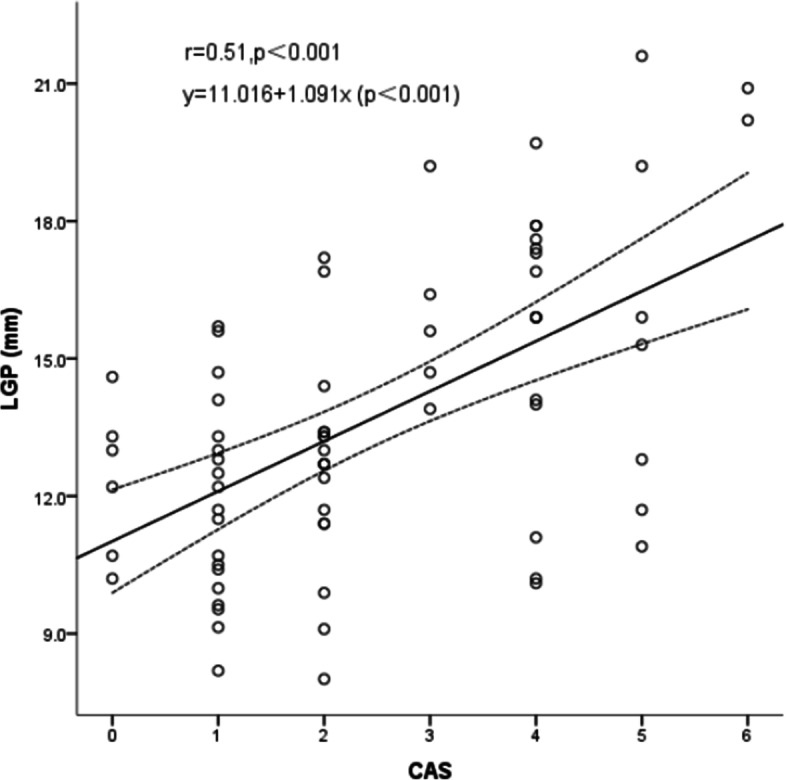
Graph showing a significant positive correlation between degree of lacrimal gland prolapse and the Clinical Activity Score (*r* = 0.51, *P* < 0.001), The linear regression equation is: y = 11.016 + 1.091x (*P* < 0.001). LGP, lacrimal gland prolapse; CAS, Clinical Activity Score

There were 29 eyes in the active group (CAS ≥3) and 43 eyes in the inactive group (CAS < 3). The mean CAS was 1.28 ± 0.69 (range, 0–2) in the inactive group and 4.21 ± 0.80 (range, 3–6) in the active group. There was a significant positive correlation between LGP severity and the CAS (r = 0.51, *P* < 0.001) (Fig. 4).

### Correlation between LGP and proptosis

**Fig. 5:**
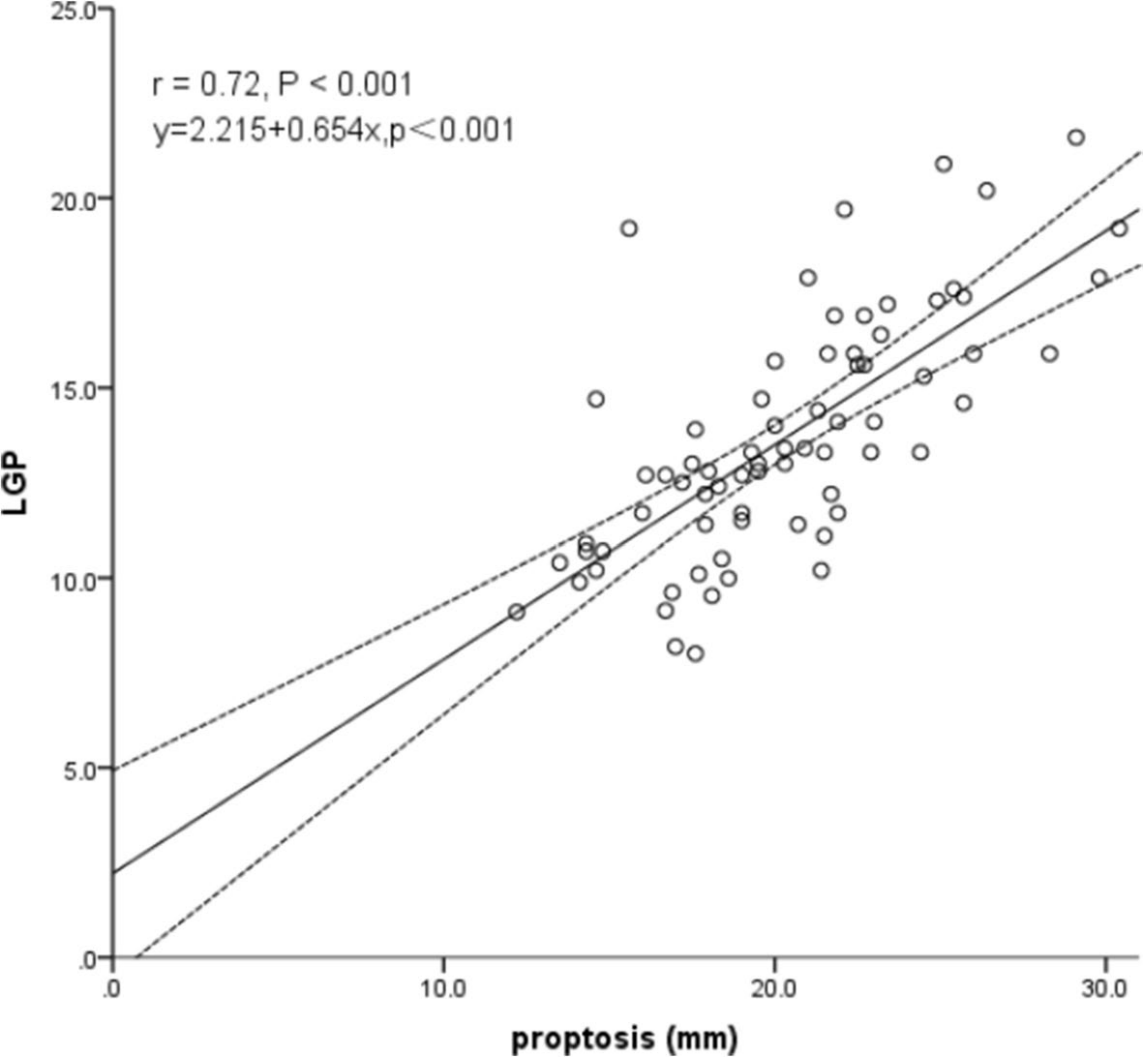
Graph showing a significant positive correlation between the degree of lacrimal gland prolapse and the amount of proptosis (*r* = 0.72, *P* < 0.001). The linear regression equation is: y = 2.215 + 0.654x (*P* < 0.001). LGP, lacrimal gland prolapse

The mean proptosis value was greater in the active group than in the inactive group (22.61 ± 4.27 vs 18.83 ± 3.02 mm) and was significantly correlated with LGP severity (*r* = 0.72, *P* < 0.001) (Fig. 5).

### Correlation between LGP severity and EOM volume

**Fig. 6:**
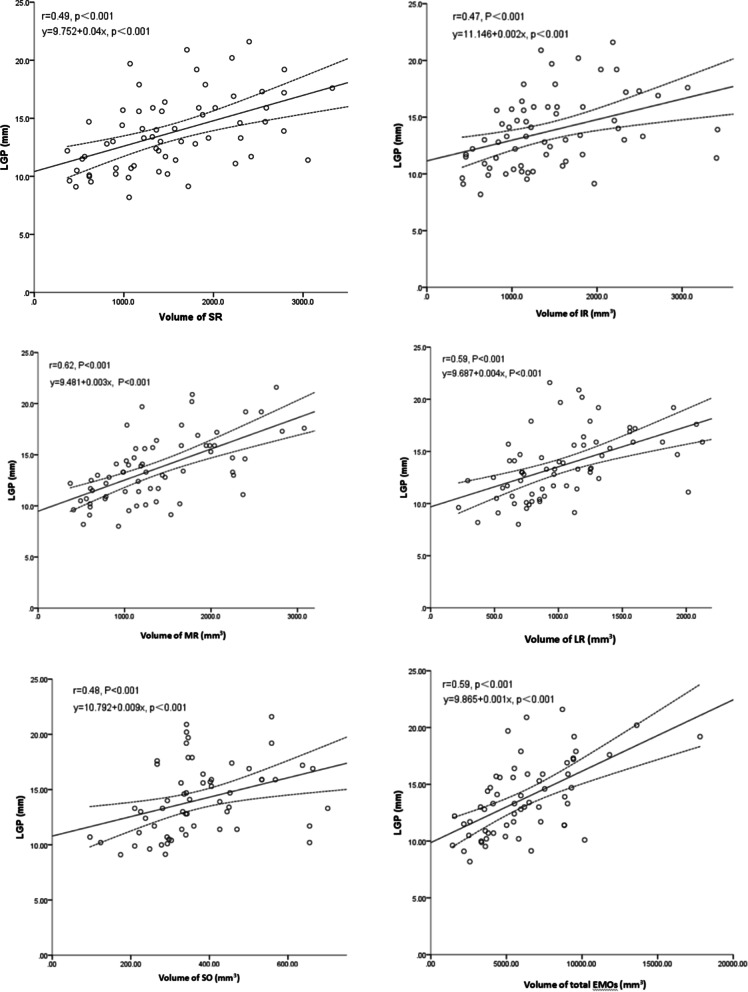
Linear regression analysis showing significant positive correlations between the degree of lacrimal gland prolapse and extraocular muscle volumes (SR, *r* = 0.49, *P* < 0.001; IR, *r* = 0.47, *P* < 0.001; LR, *r* = 0.59, *P* < 0.001; MR, *r* = 0.62, *P* < 0.001; SO, *r* = 0.48, *P* < 0.001; and all EOMs, *r* = 0.59, *P* < 0.001). IR, inferior rectus; LGP, lacrimal gland prolapse; LR, lateral rectus; MR, medial rectus; SO, superior oblique; SR, superior rectus

The mean EOM volumes for all patients were 963 mm^3^ (range, 711–1251), 1219 mm^3^ (range,884–1799), 1171 mm^3^ (range,815–1822), 1361 mm^3^ (range,827–1799), and 342 mm^3^ (range,288–449) for the lateral rectus, medial rectus, superior rectus, inferior rectus, and superior oblique muscles, respectively. The mean total EOM volume was 5669 mm^3^ (range,3764–8794). LGP severity was significantly (*P* < 0.001) positively correlated with the lateral rectus (*r* = 0.59), medial rectus (*r* = 0.62), superior rectus (*r* = 0.49), inferior rectus (*r* = 0.47), superior oblique (*r* = 0.48) muscles, and all EOMs (*r* = 0.59) (Fig. 6, Table 2).

**Table 2:** Correlations between lacrimal gland prolapse and extraocular muscle volume

	r	linear regression equation	*P*-value
MR	0.62	y = 9.481 + 0.003x	<0.01
LR	0.59	y = 9.687 + 0.004x	<0.01
SR	0.49	y = 9.752 + 0.04x	<0.01
IR	0.47	y = 11.146 + 0.002x	<0.01
SO	0.48	y = 10.792 + 0.009x	<0.01
Total	0.59	y = 9.865 + 0.001x	<0.01

*IR* inferior rectus; *LR* lateral rectus; *MR* medial rectus; *SO* superior oblique; *SR* superior rectus; *Total* sum of volume of extraocular muscles

### Correlation between LGP severity and age

There was no significant correlation between the LGP and age (r = 0.029, *p* = 0.81).

The mean ages were 40.6 ± 17.7 years (range, 21–50 years) in the young group (44 eyes) and 59.6 ± 13.4 years (range, 50–74 years) in the old group (28 patients). The mean LGP was 13.9 ± 3.4 mm in young group and 13.6 ± 2.9 in the old group. No significant difference was observed in the LGP between the young group and old group (*p* = 0.632).

### LGP cut-off value for active TAO

**Fig. 7:**
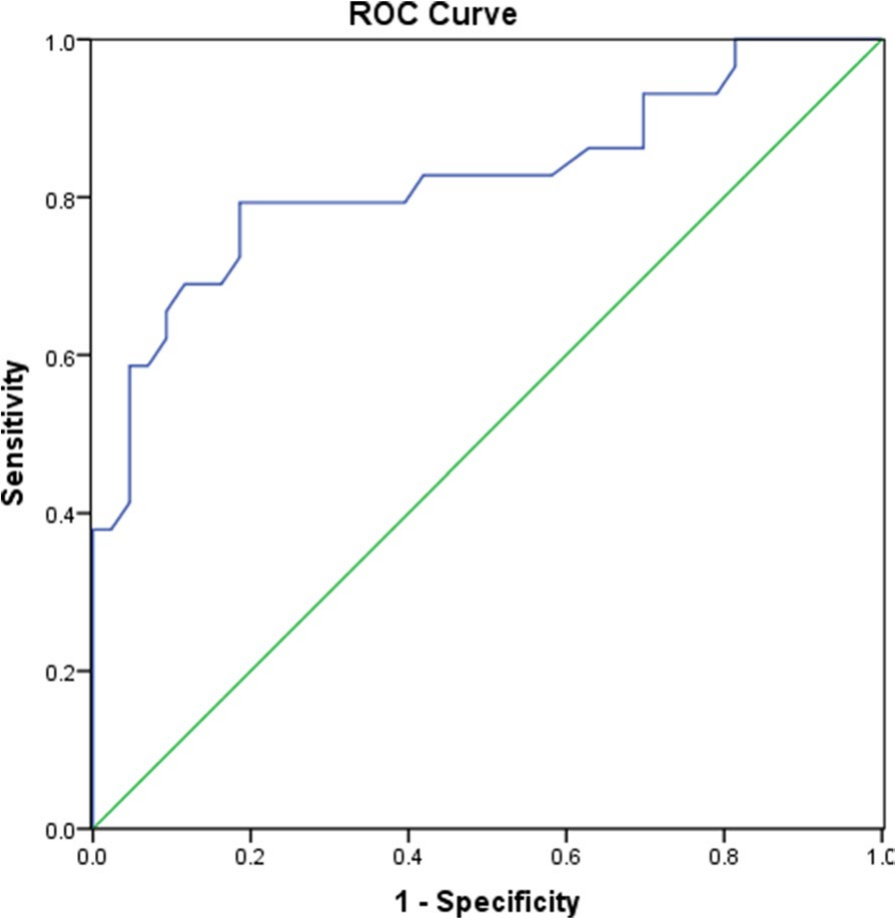
A receiver-operating characteristic curve showing the ability of lacrimal gland prolapse to discriminate active from inactive thyroid-associated ophthalmopathy. A lacrimal gland prolapse of 13.65 mm was identified as the threshold cut-off value (area under the curve, 0.824, 95% confidence interval 0.717–0.930; sensitivity, 79.3%; specificity, 81.4%)

ROC curve analysis revealed that an LGP value of 13.65 mm (area under the curve, 0.824; 95% confidence interval, 0.717–0.930) was the threshold cut-off value that differentiated active TAO (sensitivity, 79.3%; specificity 81.4%) from inactive TAO (Fig. 7).

## Discussion

This study aimed to identify a simple and convenient quantitative MRI parameter that can be used to measure the degree of LGP and determine its correlation with disease activity**.** Our findings suggest the following: (1) MRI is a simple, reproducible, and objectively quantifiable method for measuring LGP, which can reflects disease activity in patients with TAO. (2) high diagnostic accuracy can be achieved with simple imaging sequences; and (3) LGP was positively correlated with CAS, proptosis and EOM volumes, which could be a good indicator of TAO activity.

Early diagnosis and accurate staging of inflammatory activity in patients with TAO is important in terms of selecting a clinical treatment regimen and may improve the prognosis [8]. The CAS suggested by Mourits et al. [15] is a clinical tool based on redness, swelling, pain, and impaired function and is widely used for assessment of disease activity in TAO. Several studies have indicated that the CAS has high predictive value with respect to the outcome of immunosuppressive treatment [9, 16]. However, the CAS relies on the subjective judgment of clinicians and therefore has relatively poor comparability and cannot be used to quantify pathological changes. Moreover, the CAS does not directly reflect the degree of inflammation and edema in the orbital tissues. Therefore, the clinical diagnosis and treatment of TAO typically require the CAS to be combined with imaging and histological examinations in order to assess inflammatory activity.

Previous studies have used CT and MRI for quantitative assessment of the lacrimal glands to assist clinicians in the diagnosis and staging of TAO [5, 7, 17]. Imaging parameters have confirmed that the volume of the lacrimal gland is greater in patients with TAO than in healthy controls and is associated with inflammatory activity in TAO [5–7, 18]. Furthermore, Bingham et al. [6] found a marked positive correlation between the volume of the lacrimal gland and the CAS. Thus, the importance of lacrimal gland involvement in the diagnosis of TAO has been recognized.

However, calculation of lacrimal gland volume is complicated and too time-consuming for clinicians to perform in everyday clinical practice. In the present study, we found that LGP was significantly greater in patients with active TAO than in those with inactive TAO. Furthermore, there was a significant positive correlation between LGP and the CAS. ROC curve analysis indicated that an LGP value of 13.65 mm could be the optimal threshold for discriminating active and inactive TAO with high sensitivity and specificity. Therefore, LGP is a simple quantitative measurement that could be helpful in improving the diagnostic power of the CAS in the clinical evaluation of patients with TAO.

This study found positive correlations among the degree of LGP, EOM volume, and proptosis. With the development of imaging technology, changes have been observed in the lacrimal glands of patients with TAO. In 1981, Trokel et al. [19] described enlargement and enhancement of the lacrimal glands accompanied by enlarged extraocular muscles on CT images. Similarly, Nugent et al. [20] found that an anteriorly displaced lacrimal gland on CT was associated with clinical palpability and optic neuropathy in 83.3% of patients.

In view of our present findings, we suggest that the mechanism of LGP in patients with TAO involves inflammatory edema of the retrobulbar structures due to increased disease activity, resulting in enlargement and congestion of the orbital tissue. Thus, the lacrimal gland is pushed forward, leading to LGP. Studies of the hormones involved in Graves’ disease [21, 22] have demonstrated that lacrimal acinar cells contain thyroid-stimulating hormone receptors on their surface and that there is a strong correlation between the presence of thyroid antibodies and dysfunction of the lacrimal gland. Furthermore, Gagliardo et al. [11] confirmed a linear correlation between the thyrotropin receptor antibody level and the degree of LGP in patients with TAO. A pathological study also demonstrated that changes in the lacrimal gland were similar to those in the retrobulbar tissue in patients with TAO, namely, multifocal infiltration of lymphocytes and hyperplasia of adipose tissue [23]. As previously mentioned, some studies have suggested that the acinar cells in the lacrimal gland are further targets of TAO, and inflammation is the most likely cause of its enlargement [17, 24]. Therefore, we hypothesize that another reason underlying LGP is that the fibrous periosteal connections between the lacrimal gland and the orbital septum are unable to withstand enlargement of the lacrimal gland in patients with TAO.

We found no significant effect of patient age on the degree of LGP. Therefore, we believe that an impact of advancing age on LGP can be excluded. Despite the age-related increase in laxity of the lacrimal support structures, involvement of the lacrimal gland may be an independent pathogenic feature of TAO.

This study has several limitations. First, the sample size was relatively small. Nevertheless, we believe that the findings provide a basis for further studies on lacrimal gland changes in patients with TAO. Second, the imaging parameters used for the lacrimal gland were not associated with the inflammatory factors in tears and secretory function or pathological changes in the gland. This will be a topic of future research for the purpose of determining the characteristics of the lacrimal gland in the pathogenesis of TAO from an imaging perspective.

## Conclusions

This study focused on the abnormal location of the lacrimal gland in patients with TAO based on the degree of LGP as determined by MRI. These measurements appear to be a good indicator of disease activity in patients with TAO.

## Data Availability

The data that support the findings of this study are available from the corresponding author (DL) upon reasonable request.

## Abbreviations

CAS: Clinical Activity Score
CT: Computed tomography
ICC: Intraclass correlation coefficient
LGP: Lacrimal gland prolapse
MRI: Magnetic resonance imaging
ROC: Receiver-operating characteristic
TAO: Thyroid-associated ophthalmopathy
